# The War’s Recompense

**Published:** 1918-02

**Authors:** 


### DENTAL REGISTER

Vol. LXXII FEBRUARY, 1918 No. 2

### *The War's Recompense*

*YE that have faith to look with fearless eyes*
*Beyond the tragedy of a world at strife,*
*And know that out of death and night shall rise*
*The dawn of ampler life:*

*Rejoice, whatever anguish rend the heart,*
*That God has given you a priceless dower,*
*To live in these great times and have your part*
*In Freedom's crowning hour.*

*That ye may tell your sons who see the light*
*High in the Heavens—their heritage to take—*
*"I saw the powers of Darkness put to flight,*
*I saw the Morning break."*

*—From the Red Cross Magazine.*

*(The original of this verse was found on an Australian soldier who bravely fought and as nobly died. His name is as yet unknown.)*

